# The Risk of Second Primary Cancers in Prostate Cancer Survivors Treated in the Modern Radiotherapy Era

**DOI:** 10.3389/fonc.2020.605119

**Published:** 2020-11-13

**Authors:** Marie-Christina Jahreiß, Katja K. H. Aben, Mischa S. Hoogeman, Maarten L. P. Dirkx, Kim C. de Vries, Luca Incrocci, Wilma D. Heemsbergen

**Affiliations:** ^1^Department of Radiotherapy, Erasmus MC Cancer Institute, Rotterdam, Netherlands; ^2^Department of Research & Development, Netherlands Comprehensive Cancer Organization, Utrecht, Netherlands; ^3^Research Institute for Health Sciences, Radboudumc, Nijmegen, Netherlands

**Keywords:** prostate cancer, second primary cancer, survivorship, intensity-modulated radiotherapy, three-dimensional conformal radiotherapy

## Abstract

**Purpose:**

Concerns have been raised that modern intensity modulated radiotherapy (IMRT) may be associated with increased second primary cancer risks (SPC) compared to previous three-dimensional conformal radiation techniques (3DCRT), due to increased low dose volumes and more out-of-field ionizing dose to peripheral tissue further away from the target. We assessed the impact of treatment technique on SPC risks in a cohort of prostate cancer (PCa) survivors.

**Material and Methods:**

The study cohort comprised 1,561 PCa survivors aged 50–79 years at time of radiotherapy, treated between 2006–2013 (N=707 IMRT, N=854 3DCRT). Treatment details were extracted from radiotherapy systems and merged with longitudinal data of the Netherlands Cancer Registry to identify SPCs. Primary endpoint was the development of a solid SPC (excluding skin cancer) in peripheral anatomical regions, i.e. non-pelvic. Applied latency period was 12 months. SPC rates in the IMRT cohort (total cohort and age subgroups) were compared to 1) the 3DCRT cohort by calculating Sub-Hazard Ratios (sHR) using a competing risk model, and 2) to the general male population by calculating Standardized Incidence Ratios (SIR). Models were adjusted for calendar period and age.

**Results:**

Median follow-up was 8.0 years (accumulated 11,664 person-years at-risk) with 159 cases developing ≥1 non-pelvic SPC. For IMRT vs 3DCRT we observed a significantly (*p*=0.03) increased risk (sHR=1.56, 95% Confidence Interval (CI) 1.03–2.36, corresponding estimated excess absolute risk (EAR) of +7 cases per 10,000 person-years). At explorative analysis, IMRT was in particular associated with increased risks within the subgroup of active smokers (sHR 2.94, *p*=0.01). Within the age subgroups 50–69 and 70–79 years, the sHR for non-pelvic SPC was 3.27 (*p*=0.001) and 0.96 (*p*=0.9), respectively. For pelvic SPC no increase was observed (sHR=0.8, *p=*0.4). Compared to the general population, IMRT was associated with significantly increased risks for non-pelvic SPC in the 50–69 year age group (SIR=1.90, *p*<0.05) but not in the 70–79 years group (SIR=1.08).

**Conclusion:**

IMRT is associated with increased SPC risks for subjects who are relatively young at time of treatment. Additional research on aspects of IMRT that may cause this effect is essential to minimize risks for future patients receiving modern radiotherapy.

## Introduction

Radiotherapy (RT) is an important treatment modality in cancer treatment and intensity-modulated radiotherapy (IMRT) is one of its most important recent developments. It is characterized by a highly conformal dose delivery to the tumor and hence improved sparing of normal tissue. This requires a longer beam-on time and results in larger low dose volumes and more out-of-field ionizing dose to peripheral healthy tissue further away from the target (1, 2). It is therefore increasingly recognized that modern RT might result in excess DNA damage in peripheral tissues, potentially increasing the risk of SPC, especially for young patients with favorable survival (3–5). To date, there are insufficient clinical data to draw firm conclusions about the impact modern RT has on SPC risks (6, 7).

In the Netherlands and worldwide, the prostate cancer (PCa) patient population was one of the first groups for whom IMRT was introduced (8). PCa is one of the most common cancer diagnoses with a growing population of long-term survivors (9). Many previous cohort studies in the 3DCRT era have addressed SPC risks in PCa survivor cohorts. They consistently reported limited excess SPC risks for mainly bladder and rectal cancer (6, 7, 9, 10). The impact of IMRT dose distributions on SPC risks is a topical research question, addressed in many recent model studies, evaluating risks for different age categories and different IMRT features. Their conclusions vary from hypothesized limited excess risks to a two- to threefold increase in radiation-induced SPCs (1, 2, 11). Therefore there is a need for large cohort studies with clinical data and sufficient follow-up to further address this issue.

The aim of the present study was to assess the risk of developing a SPC in a PCa survivor cohort, comparing an IMRT cohort with the Dutch general population (external reference group) and with a 3DCRT cohort (internal reference group), treated in the same era and at the same institute for localized PCa. We hypothesized that IMRT is associated with excess out-of-field (i.e. non-pelvic) SPC risks due to increased peripheral dose levels.

## Methods

### Study Design and Participants

This retrospective cohort study consisted of 1,561 PCa survivors, previously treated with RT for localized PCa at the Erasmus Medical Center, Rotterdam, The Netherlands between 2006–2013, aged between ≥50–<80 years at time of treatment. RT was planned and delivered with either IMRT or 3DCRT. IMRT was gradually introduced during the period 2007–2010. Exclusion criteria for this study were the presence of metastatic disease, previous RT to pelvis areas and simultaneous treatment for other tumors. The study protocol was reviewed by the Medical Ethical Committee of the Erasmus Medical Center (EMC 1812730) and permission was obtained for retrospective anonymized data collection, in accordance with local and national regulations.

### Radiotherapy Protocol

The prescribed dose to the prostate was 72 Gy or 78 Gy in daily fractions of 2 Gy; from June 2010 onwards 78 Gy became the standard prescription dose for intermediate to high-risk disease. The prescribed dose to the Seminal Vesicles (SVs) varied between 0–78 Gy, depending on the estimated probability of SV involvement and slightly changing guidelines over time (12, 13). Elective lymph node irradiation was not applied. IMRT beam arrangements typically included a 7-field technique and 3DCRT was delivered with a 3-field technique (two lateral and one anterior-posterior field). The planning target volume was the prostate (+/− SV) plus 10 mm margin in case of off-line setup verification, and 5–7 mm in case of on-line setup verification. A full bladder protocol was applied. Offline setup verification was performed on bony anatomy during the first three days and then weekly; online verification was performed with daily tracking of the prostate position using intra-prostatic implanted fiducial markers. The image-guidance procedures were performed with planar megavoltage (MV) portal imaging, or, for 10 MV IMRT with a combination of planar MV and planar kilovoltage (kV) imaging. For offline MV imaging, additional larger pelvic imaging field were applied incidentally, when the treatment field did not contain sufficient information for the verification procedure.

### Data Collection

We collected data from the electronic patient files and RT systems at the Erasmus MC and from the nationwide Netherlands Cancer Registry (NCR). This included patient and tumor characteristics, smoking status, adjuvant hormonal therapy prescription, and details of the RT course. Information regarding SPC diagnosis, vital status, date of death (if applicable), and date of emigration was obtained from the NCR. Linkage was performed based on date of birth and postal code at time of treatment. In six cases no match could be made. For these patients the SPC and vital status information was extracted from the electronic patient files. All cancers in the NCR are coded according to The International Classification of Disease for Oncology (ICD-O-3), and for this study were converted to ICD-10.

### SPC Endpoints

We defined time at risk for SPC from 1 year after start of RT onwards. For SIR calculations we used 1 year after diagnosis as a surrogate since start RT is not captured as a required data field in the NCR database. The following cancer sites were evaluated (further details in **Supplementary File 1**): a) all solid malignant neoplasms (C00–C80) except mesothelioma, skin cancer and prostate cancer, b) solid pelvic SPCs c) solid non-pelvic SPCs (primary endpoint), d) solid cancers per anatomic region (pelvis, thorax, abdomen), and e) solid separate (grouped) cancer sites – provided that sufficient events were observed.

### Statistical Analysis

Standardized incidence ratios (SIRs) were calculated in order to determine the risk of developing a SPC among PCa survivors in comparison with the Dutch general population. The SIR was computed by dividing the observed cancer cases in this study by the expected cancer cases in the Dutch general population based on age-, gender-, and calendar specific cancer incidence rates retrieved from the NCR. For SIR calculations, patients with no match, patients with <1-year follow-up, and patients diagnosed with PCa before the year 2005, i.e. with larger interval between first diagnosis and RT, were excluded (n=13), leaving 1,548 subjects for analysis. All subsequent SPCs after PCa diagnosis were considered in this analysis up to December 31^st^ 2019. SIRs were calculated for all defined SPC endpoints for the IMRT cohort, 3DCRT cohort and total cohort. Additionally, SIRs were calculated for different patient age groups (50–69 years and 70–79 years). The SIR analysis and 95% Confidence Intervals (CI) were calculated using SAS version 9.2 (SAS Institute Inc., Cary, NC, USA).

For the comparison of the exposed IMRT cohort versus the reference 3DCRT cohort, competing risk analysis was carried out using the Fine and Gray method for estimating relative risk [sub Hazard Ratios (sHRs)] (14). Only the first SPC after PCa diagnosis was considered. We adjusted all models for age at RT and calendar period. Cumulative incidences of SPCs were estimated in the presence of death and non-solid SPC as competing risk. SPCs occurring within 3 months of one another were considered as synchronous and both included in the analysis. Follow-up duration was defined as time since start RT until the date of diagnosis of SPC, date of death, date of emigration or end of follow-up or end of study (whichever came first). All patients were censured after 11 years of follow-up to adjust for differences in maximum follow-up between the two groups, as IMRT was later introduced. End of study was December 2019. Significance was set at p <0.05. The competing risk analysis was conducted using Stata version 14 (STATA Corp., Texas, USA).

## Results

### General

Baseline and treatment-related characteristics are displayed in **Table 1**. Out of the 1,561 PCa survivors, 707 received IMRT and 854 received 3DCRT. Median age at follow-up was 71 years (IQR 64–74) for the IMRT group and 71 years (IQR 66–75) for the 3DCRT group. As indicated in **Table 1**, the IMRT cohort differed significantly from the 3DCRT cohort with respect to most of the reported characteristics. In particular, they received much more often 78 Gy (98 vs 56% in the 3DCRT group). With respect to treatment, the proportion of IMRT patients receiving adjuvant hormonal therapy (57 vs 48%) and the proportion with inclusion of the seminal vesicles (83 vs 77%) was larger as well. Furthermore, the applied photon beam energy differed, and IMRT patients were more often treated with an online protocol which involved smaller safety margins and daily imaging; daily imaging was however not performed with advanced 3D cone beam imaging but for most cases with 2D treatment fields (as described in the *Methods* section). Distribution of smoking in the IMRT and 3DCRT were very similar with 18% smoking at time of treatment. Overall survival of the cohort was 62% (IMRT) and 60% (3DCRT) at 10 years (p=0.8).

**Table 1:** Baseline Characteristics of the IMRT and 3DCRT PCa patient cohort (N=1,561).

	IMRT (n=707)		3DCRT (n=854)		p-value
	n	%	n	%	
**Patient **					
**Birth cohort**					
**<1935**	99	14.0%	278	32.6%	<0.01
**1935–1940**	281	39.7%	276	32.3%	
**>1940**	327	46.3%	300	35.1%	
**Age at radiotherapy**					
**50–69**	286	40.5%	392	45.9%	0.031
**70–79**	421	59.5%	462	54.1%	
**Smoking status at time of RT**					
**Never Smoker**	219	31.0%	276	32.3%	0.3
**Past Smoker**	162	22.9%	190	22.2%	
**Active Smoker**	125	17.7%	150	17.6%	
**Not Reported**	201	28.4%	238	27.9%	
**Interval diagnosis-RT**					
**1–6 months**	617	87%	773	91%	<0.01
**>6–12 months**	33	5%	51	6%	
**>12 months**	57	8%	30	3%	
**Tumor**					
**Previous cancer diagnosis**					
**No**	662	93.6%	798	93.4%	0.9
**Yes**	45	6.4%	56	6.6%	
**PCa Risk group**					
**Low-Intermediate**	228	32.2%	354	41.5%	<0.01
High*	479	67.8%	500	58.5%	
**Treatment**					
**Adjuvant Hormonal Therapy**					
**Yes**	403	57.0%	412	48.2%	0.023
**No**	304	43.0%	442	51.8%	
**Calendar Period RT**					
**2006–2009**	83	11.7%	673	78.8%	<0.01
**2010–2012**	624	88.3%	181	21.2%	
**Dose Prostate (P)**					
**72 Gy**	16	2.3%	373	43.7%	<0.01
**78 Gy**	691	97.7%	481	56.3%	
**Dose Seminal Vesicles (SV)**					
**0 Gy**	123	17.4%	195	22.8%	<0.01
**50–78 Gy**	584	82.6%	659	77.2&	
**Image-Guidance**					
**Offline (bony anatomy)**	129	18.2%	854	100%	<0.01
**Online (prostate)**	578	81.8%	0	0%	
**Energy Megavolts (MV)**					
**10**	449	63.5%	4	0.5%	<0.01
**18**	258	36.5%	318	37.2%	
**23**	0	0%	532	62.3%	

*T3 or Gleason >7 or PSA >20 µ/L.

During follow-up, 225 survivors developed a solid SPC in the total cohort with 11,664-person-years, excluding the latency of 1 year calculated from start RT; excluding the latency of 1 year calculated from date of diagnosis (for the SIR analysis), 233 survivors had developed a solid SPC (**Tables 2****–****4**). This results in an overall crude SPC rate of 19.3 events of a first SPC per 10,000 person-years. In the IMRT cohort (4,738 person-years), 99 solid SPCs were observed, which is a crude incidence rate of 25.1 SPCs per 10,000 person-years. In the 3DCRT cohort (126 SPCs in 6,926 person-years) the crude incidence rate was 19.2 SPCs per 10,000-person years (**Table 4**).

**Table 2:** Standardized Incidence Ratios (SIRs) for the total cohort compared to the general Dutch male population, adjusted for age and calendar year.

Second Tumor Site	Observed (n)	Expected (n)	SIR (95% CI)
**All solid (excluding skin)**	233	254.3	0.92 (0.80–1.04)
**All solid non-pelvis**	168	140.9	**1.19 (1.02–1.39)**
**Anatomical Regions**			
**Chest**	73	62.8	1.16 (0.91–1.46)
**Lung & bronchus**	49	49.4	0.99 (0.73–1.31)
**Abdomen**	73	60.7	1.20 (0.94–1.51)
**Esophagus**	16	8.8	**1.82 (1.04–2.95)**
**Stomach**	3	4.9	0.61 (0.13–1.79)
**Colon**	31	33.2	0.93 (0.63–1.22)
**Pancreas**	11	7.8	1.41 (0.70–2.52)
**Kidney, Renal Pelvis and Ureter**	18	11.7	1.54 (0.91–2.43)
**Pelvis**	70	50.2	**1.39 (1.09–1.76)**
**Bladder & Urethra**	50	33.4	**1.50 (1.11–1.97)**
**Rectum & Rectosigmoid**	21	15.3	1.37 (0.85–2.10)
**Sub-sites**			
**Urinary Tract**	68	45.8	**1.48 (1.15–1.88)**
**Gastrointestinal**	95	78.1	1.22 (0.98–1.49)
**Central Nervous System**	5	2.6	1.92 (0.62–4.49)
**Unknown**	7	5.3	1.32 (0.53–2.72)
**Age Groups**			
**Patients aged 50–69 years**			
**All solid SPC**	101	77.3	**1.31 (1.06–1.59)**
**All solid SPC non-pelvis**	74	54.4	**1.36 (1.07–1.71)**
**Patients aged 70-79 years**			
**All solid SPC**	139	122.6	1.13 (0.95–1.34)
**All solid SPC non-pelvis**	94	86.4	1.09 (0.88–1.33)

Observed and expected reflect number of observed and expected survivors experiencing the SPC event of interest. For SPC sub-sites, all experienced SPCs are taken into consideration. SIR=observed/expected. Evaluation period is diagnoses + 1 year up to end of exposure (end of observation period is 31-12-2019). Grouping of regions and ICD10 codes is reflected in **Supplementary File 1**.

Bold numbers represent significantly elevated SIRs (p < 0.05).

**Table 3:** Standardized Incidence Ratios (SIRs) for the IMRT and 3DCRT cohort compared to the general Dutch male population, adjusted for age and calendar year.

Second Tumor Site	IMRT (n=697)			3D-CRT (n=851)		
	Observed (n)	Expected (n)	SIR (95% CI)	Observed (n)	Expected (n)	SIR (95% CI)
**All solid (excluding skin)**	101	96.4	1.05 (0.85–1.27)	132	158.1	0.83 (0.70–0.99)
**All solid non-pelvis**	73	53.4	**1.37 (1.07–1.72)**	95	87.4	1.09 (0.88–1.33)
**Anatomical Regions**						
**Chest**	33	23.6	1.40 (0.96–1.96)	40	39.1	1.02 (0.73–1.39)
**Lung & bronchus**	23	18.4	1.25 (0.79–1.88)	26	30.9	0.84 (0.55–1.23)
**Abdomen**	24	23.5	1.02 (0.65–1.52)	49	37.2	1.32 (0.97–1.74)
**Esophagus**	8	3.5	2.29 (0.99–4.50)	8	5.2	1.54 (0.66–3.03)
**Stomach**	1	1.8	0.56 (0.01–3.10)	2	3.3	0.61 (0.07–2.19)
**Colon**	9	12.7	0.71 (0.32–1.35)	22	20.5	1.07 (0.67–1.62)
**Pancreas**	2	2.9	0.69 (0.08–2.49)	9	4.9	1.84 (0.84–3.49)
**Kidney, Renal Pelvis and Ureter**	8	4.4	1.82 (0.78–3.58)	10	7	1.43 (0.69–2.63)
**Pelvis**	31	19	**1.63 (1.11–2.32)**	39	31.2	1.25 (0.89–1.71)
**Bladder & Urethra**	20	12.5	1.6 (0.98–2.47)	30	21	1.43 (0.96–2.04)
**Rectum & Rectosigmoid**	12	5.8	**2.07 (1.07–3.61)**	9	9.4	0.96 (0.44–1.82)
**Sub-sites**						
**Urinary Tract**	27	17.5	1.54 (1.02–2.24)	41	28.4	**1.44 (1.04–1.96)**
**Gastrointestinal**	37	30	1.23 (0.87–1.70)	58	48.2	1.20 (0.91–1.56)
**Central Nervous System**	4	1	**4.00 (1.09–10.24)**	1	1.7	0.59 (0.01–3.28)
**Unknown**	4	1.7	2.35 (0.64–6.02)	3	3.4	0.88 (0.18–2.58)
**Age Groups**						
**Patients aged 50–69 years**						
**All solid SPC**	48	26.1	**1.84 (1.36–2.44)**	53	51.6	1.03 (0.77–1.34)
**All solid SPC non-pelvis**	35	18.4	**1.90 (1.32–2.65)**	39	36.1	1.08 (0.77–1.48)
**Patients aged 70–79 years**						
**All solid SPC**	56	50.3	1.11 (0.84–1.45)	83	72.2	1.15 (0.92–1.43)
**All solid SPC non-pelvis**	38	35.3	1.08 (0.76–1.48)	56	51.3	1.09 (0.82–1.42)

Observed and expected reflect number of observed and expected survivors experiencing the SPC event of interest. For SPC sub-sites, all experienced SPCs are taken into consideration. SIR=observed/expected. Evaluation period is diagnoses + 1 year up to end of exposure (end of observation period is 31-12-2019). Grouping of regions and ICD10 codes is reflected in **Supplementary File 1**.

Bold numbers represent significantly elevated SIRs (p < 0.05).

**Table 4:** Crude incidence rates (per 10,000-person years) and estimated subHazard Ratios by Gray and Fine method (with adjustment for age and calendar year at time of radiotherapy) for IMRT versus the reference group 3DCRT.

Subsite (ICD10 code)	IMRT		3DCRT		Fine & Gray Model	
	n with SPC	Incidence rate	n with SPC	Incidence rate	sHRs (95% CI)	p-value
**All solid (except skin) C00–C80**	99	20.90	126	13.01	1.23 (0.88–1.76)	0.220
**Solid SPC non-pelvis**	74	15.62	85	12.27	1.56 (1.03–2.36)	**0.034**
**Age at RT**						
**50–69 years**	37	18.93	29	8.49	3.27 (1.65–6.46)	**0.001**
**70–79 years**	37	13.29	56	15.96	0.96 (0.59–1.59)	0.887
**Smoking Status at RT**						
**Never smoker**	17	10.79	24	10.47	0.80 (0.32–1.98)	0.631
**Past smoker**	21	20.66	30	19.60	0.94 (0.43–2.06)	0.875
**Active smoker**	25	33.33	15	12.94	2.94 (1.28–6.76)	**0.011**
**Not reported**	11	7.88	16	8.22	1.20 (0.42–3.40)	0.734
**SPC within anatomical regions**						
**Chest**	30	6.33	36	5.20	1.21 (0.65–2.26)	0.548
**Abdomen**	26	5.48	42	6.06	1.13 (0.59–2.16)	0.719
**Pelvis**	27	5.70	40	5.77	0.79 (0.41–1.47)	0.437
**SPC by tumor site**						
**Urinary tract total**	23	4.85	37	5.34	0.72 (0.35–1.49)	0.380
**Bladder, urethra**	17	3.59	29	4.19	0.56 (0.27–1.18)	0.128
**Kidney, renal pelvis, ureter**	8	1.69	6	0.87	3.90 (0.64–23.80)	0.140
**Gastrointestinal total**	34	7.18	53	7.65	1.07 (0.62–1.86)	0.802
**Rectum, rectosigmoid**	10	2.11	9	1.30	2.41 (0.76–7.64)	0.135
**Colon**	10	2.11	17	2.45	0.82 (0.31–2.15)	0.688
**Oesophagus & Stomach**	9	1.90	13	1.88	1.30 (0.41–4.19)	0.655
**Pancreas, liver, biliary tract**	6	1.27	14	2.02	0.57 (0.20–1.61)	0.287
**Lung & Bronchus**	21	4.43	24	3.46	1.19 (0.57–2.48)	0.636
**Soft tissue/sarcoma**	2	0.42	2	0.29	1.20 (0.05–31.84)	0.914

Numbers reflect observed number of survivors experiencing the SPC event of interest. For SPC sub-sites, only the first SPC is taken into consideration. Grouping of regions and ICD10 codes is reflected in **Supplementary File 2**.

Bold numbers represent significantly elevated SIRs (p < 0.05).

### Comparison to the General Population

The estimated SIR (95% confidence interval) for any solid SPCs (excluding skin, mesothelioma, and prostate) was 0.92 (0.80–1.04) in the total cohort (**Table 2**), 1.05 (0.85–1.27) in the IMRT cohort and 0.83 (0.70–0.99) in the 3DCRT cohort (**Table 3**). For non-pelvic tumors the estimated SIR for solid SPCs (excluding skin, mesothelioma, and prostate) was 1.37 (1.07–1.72) in the IMRT cohort and 1.09 (0.88–1.33) in the 3DCRT cohort (**Table 3**).

### Comparison of IMRT With 3DCRT

**Figure 1:**
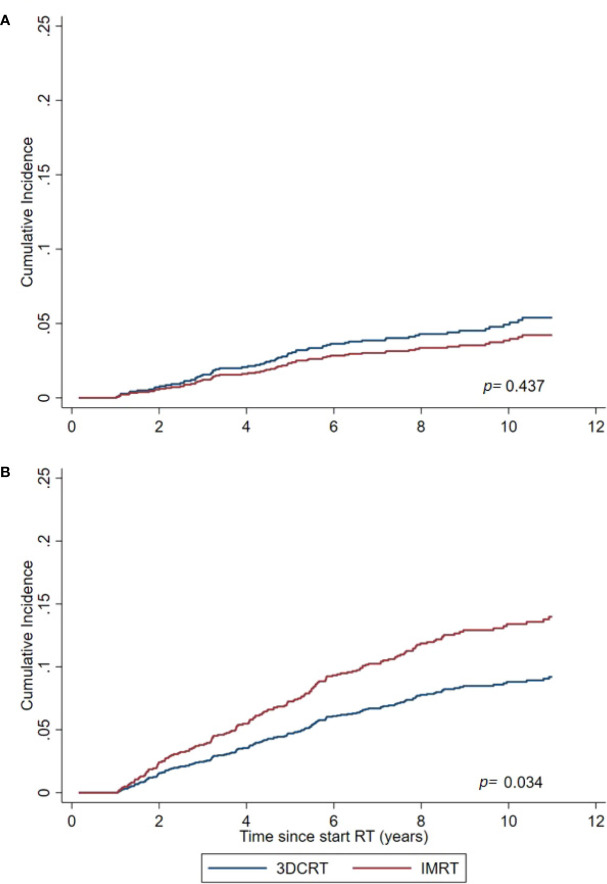
Cumulative Incidence of solid SPCs for the IMRT and 3DCRT cohort, as estimated by the Fine and Gray model adjusted for calendar period and age. **(A)** For SPCs in the pelvic region. **(B)** For non-pelvic SPCs (primary endpoint).

For the total cohort the adjusted sHR (95% confidence interval) (IMRT vs 3DCRT) for developing a solid SPC outside the pelvis was 1.56 (1.03–2.36, p=0.034) (**Table 4**, **Figure 1**). For all SPCs regardless anatomic region, this was 1.23 (0.88–1.76, p=0.220). For pelvic tumors this was 0.79 (0.41–1.47, p=0.437).

### Sensitivity Analysis

We evaluated in a sensitivity analysis whether other treatment-related factors had an impact as well, since the IMRT and 3DCRT cohort differed with respect to seminal vesicle dose, prostate dose, adjuvant hormonal treatment prescription, and using online or offline setup protocol (with different safety margins). Note that for online imaging, no 3D cone beam CT was involved (as described in the *Methods* section). For that purpose, we added each factor to our baseline multivariable model for the main endpoint of non-pelvic SPC. Results are summarized in **Supplementary File 2**, including estimated sHRs of these additional factors and the corresponding sHRs for IMRT vs 3DCRT within each alternative model. It shows that none of the other treatment factor had an impact, and estimated SHRs for IMRT vs 3DCRT within each alternative model remained very similar to the baseline model estimate. In addition, we also repeated our main analysis for the 78 Gy subgroup only, since the IMRT and 3DCRT group show the largest difference with respect to this treatment characteristic. As shown in **Supplementary File 2**, the estimated sHR remained again very similar (1.53 versus 1.56 for the total group). The corresponding p value was 0.055 (IMRT vs 3DCRT, 78 Gy subcohort only).

As described in the following paragraphs, smoking status was associated with SPC risks, but the distribution of smoking status was equal between the RT technique groups.

### Age Subgroups

**Figure 2:**
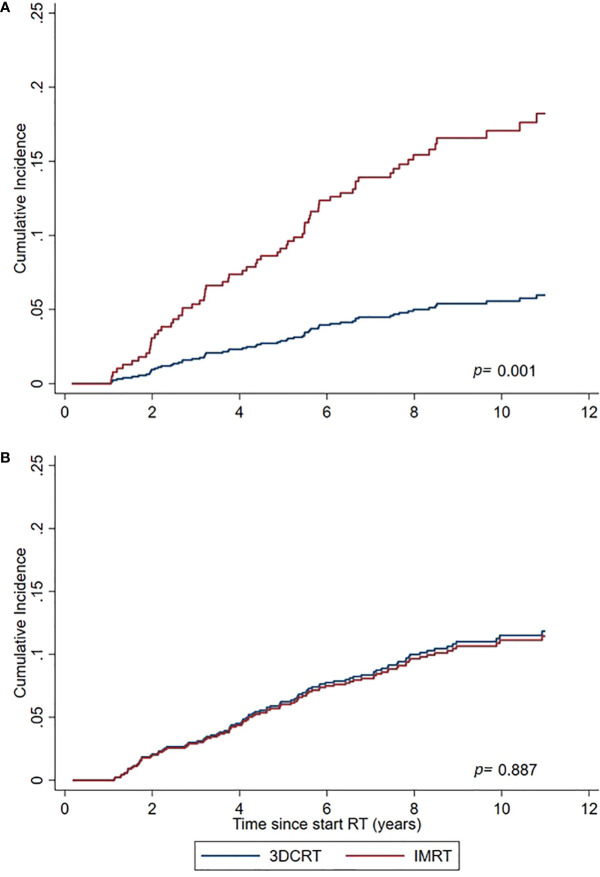
Cumulative Incidence of non-pelvic SPCs for IMRT and 3DCRT subgroups, as estimated by the Fine and Gray model adjusted for calendar period and age. **(A)** PCa survivors aged 50–69 years at time of treatment. **(B)** PCa survivors aged 70–79 years at time of treatment.

In PCa survivors aged 50–≤ 69 years at time of treatment, IMRT was associated with a significant excess SPC risk compared to 3DCRT (sHR=3.27 (1.65–6.46) and compared to the general population (SIR=1.31 (1.06–1.59) whereas for the elderly aged 70–79 years no increased risks were observed (**Tables 3**, **4**, **Figure 2**).

### Smoking Subgroups

**Figure 3:**
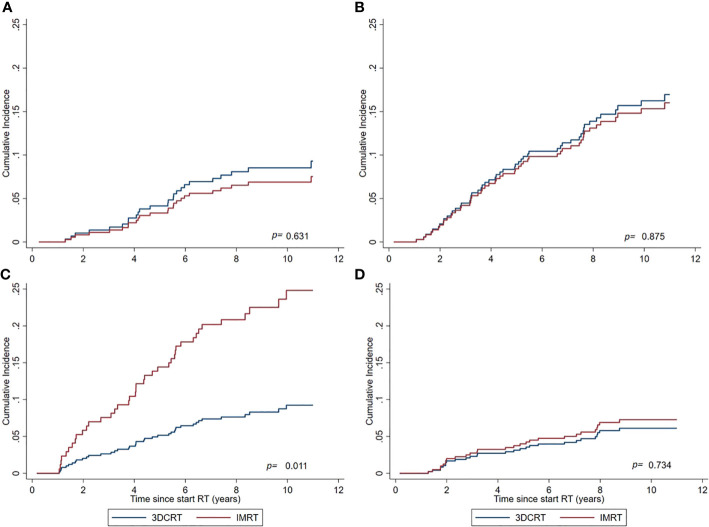
Cumulative Incidence of non-pelvic SPCs for IMRT and 3DCRT subgroups, as estimated by the Fine and Gray model adjusted for calendar period and age. **(A)** Never smokers. **(B)** Previous smokers at time of treatment. **(C)** Active smokers at time of treatment. **(D)** Smoking status not reported in patient files.

In general, active smoking at time of treatment was significantly associated with increased SPC risks in the total cohort regardless RT technique (sHR=1.67 (1.16–2.41) for active smokers vs other smoking categories). Comparing IMRT with 3DCRT within the subgroup of active smokers, the sHR for non-pelvic SPC was 2.94 (1.28–6.76), whereas no significant differences between IMRT and 3DCRT were observed for the remaining smoking categories (**Table 4**, **Figure 3**). The observed interaction between treatment technique and active smoking yes/no was significant (p<0.01). For the endpoint pelvic SPC, no difference was observed for IMRT vs 3DCT within the active smoker subgroup (sHR=1.79 (0.48–6.70). Evaluating specific frequent SPC endpoints known to be related to smoking (lung, bladder), we observed no significant differences between IMRT and 3DCT. A comparison with the general population within smoking categories was not possible because smoking status is not registered in the NCR.

### Other SPC Endpoints

Regarding anatomical regions and specific tumor types, most prominent differences between IMRT and the internal and external reference populations were found for bladder/urethra, kidney/renal pelvis/ureter and rectum/rectosigmoid (**Tables 3**, **4**). Comparing 3DCRT to the general population, only excess cancer risks (p<0·05) were observed for the genitourinary tract, whereas for IMRT excess cancer risks (p<0·05) were observed for many SPC endpoints (**Table 4**): all solid, non-pelvic pelvic, rectosigmoid, and genitourinary. For the combined RT cohort, significant excess risks were observed for the SPC endpoints solid non-pelvis, esophagus, pelvis, bladder, and urinary Tract, and for the age subgroup 50–69 years (all solid and non-pelvis) (**Table 2**).

### Excess Absolute Risk (EAR)

Based on the results of the Fine and Gray model, the estimated EAR for non-pelvic SPC (per 10,000 person-years) for IMRT is +7 SPC cases (95% CI 1–16) in addition to the baseline risk of 12 cases with 3DCRT. For the age subgroup 50–69 years the estimated EAR is +19 cases with a quite large confidence interval (5–46), in addition to the baseline risk of 8 cases with 3DCRT. If we take the ratio of the estimated SIRs for IMRT and 3DCRT as an alternative estimate of the relative SPC risks and associated AERs, the estimated AERs for non-solid SPC (IMRT vs 3DCRT) are smaller: +3 cases for IMRT – total group, and +7 for IMRT – 50–69 years.

## Discussion

To our knowledge, this is the first study evaluating the impact of IMRT on SPC risks in a large patient population with full details on their treatment for localized PCa, comparing risks to both the general population and an internal 3DCRT reference group. We observed a significantly increased risk for the primary endpoint of non-pelvic SPC, in particular for the subgroups of age 50–69 years and active smokers. The IMRT population did differ from the 3DCRT population with respect to the prescribed dose level (more often 78 Gy) and prescription of adjuvant hormonal therapy, however, at the performed sensitivity analysis we demonstrated that these factors did not affect our estimates of the main results.

Previously, Journy et al. (15) assessed a large prostate cancer cohort from the SEER (Surveillance, Epidemiology, and End Results) registry database, with patients aged >65–<85 years treated with either IMRT or 3DCRT. For the endpoint of solid tumors in 5-years survivors, they observed no significant differences between IMRT and 3DCRT, which is in rough agreement with our results for the age group >69 years. Buwenge et al. (16) compared PCa patients treated with 3DCRT (n=538) to PCa patients treated with IMRT (n=1988). They reported elevated SPC risks in the pelvic region for IMRT treated with nodal irradiation. Since our population was not subject to nodal irradiation, a valid comparison to our results is not possible. Zelefsky et al. (17) studied SPC rates in a PCa population treated with IMRT (n=897) or brachytherapy (n=413) with a median of 8y follow-up, comparing SPC risks between the modalities and each modality with the general population, and reported no excess risks in the IMRT group compared to the general population and the brachytherapy group for the overall SPC endpoint and for infield and out of field SPC (excluding skin cancer). For the IMRT vs brachytherapy comparison, they report a 10y estimated cumulative SPC incidence of 16 vs 12% (p=0.3) They included no 3DCRT group for comparison. Furthermore, the brachytherapy group contained a subgroup treated with both brachytherapy and additional IMRT to the prostate and seminal vesicles. Xiang et al. (5) compared SPC rates between IMRT, 3DCRT, and proton cohorts for various tumor sites with a median follow-up of 5 years, and reported for PCa favorable SPC rates after proton therapy and no significant impact of IMRT compared to 3DCRT.

In the present study we used data from the general male population for the calculation of background SPC risks in the IMRT and 3DCRT cohort which is a broadly applied approach in SPC research (17–20). An alternative approach often used in prostate cancer cohorts, is to compare SPC rates between radiotherapy and other treatment modalities such as surgery (7). Reported results on elevated SPC risks of these two types of approaches are roughly in agreement, identifying increased SPC risk after PCa radiotherapy for bladder cancer and rectal cancer, and in some studies also for other types of cancer in or close to the pelvic area such as soft tissue carcinoma and colon cancer (7, 9, 19). We are currently working on a separate project, using national cancer registry data, in which we compare SPC patterns between several PCa treatment modalities (external beam radiotherapy, brachytherapy, surgery, active surveillance) for different calendar periods in order to estimate additional risks of external beam radiotherapy versus other treatment options including possible time trends related to the introduction of modern radiotherapy.

Age is an important factor for the risk of developing a radiation-induced cancer, as previously demonstrated in the atomic bomb Life Span studies, as well as in other cohort studies on the impact of radiation on cancer risks (3, 21–23). In line with these observations from epidemiological studies, our study results showed that relative young patients (<69) are at increased risk for a (radiation-induced) SPC using IMRT whereas older patients have no or limited risks. In a recent study of Krasnow et al. (24), excess SPC risks (bladder, rectum) for different age categories of PCa cancer survivors were estimated and they reported highest Hazard Ratios (HR) for survivors aged < 65 years at time of treatment (HR of 1.7), limited risks for age category >75 year (HR of 1.1), and a HR of about 1.4 for the categories in between. This emphasizes the importance of distinguishing between the risk older PCa patients receiving IMRT are exposed to, to that of relatively younger patients.

In this study we applied a latency period of 1 year. In a cohort study of Arnold et al. (18), evaluating SPCs after RT in the pelvic region in females (for cervical cancer), excess risks were evaluated for different follow-up periods. They observed no excess risks for 0.5–1 years, and they did report excess risks for the periods 1–5 years, 5–10 years, and 10+ years. Several studies showed in particular an increased risk for radiation-induced SPC for the period after 10 years of follow-up (9, 10). However, in a PCa population with most cases aged between 60–80 years, especially excess SPC risks in the first 10 years are important since they will have eventually have the most impact on survival rates.

Bladder cancer is the most commonly reported radiation-induced SPC after RT for PCa. A recent study of Moschini et al. (25), comparing RT with prostatectomy, showed increased rates of bladder cancer from 1year follow-up onwards. Wallis et al. (7) showed that studies with and without a latency period of 5 years observed similar excess risks for bladder cancer (7). We observed increased bladder cancer rates compared to the general population (SIR=1.5 for the combined cohort), with no differences between IMRT and 3DCRT.

For rectal cancer we observed no increased risks with respect to the general population in the 3DCRT group whereas for IMRT a significant increase was observed. Based on literature, for both groups an increase was expected (26–28). However, Moschini et al. (25) observed no increased risks for rectal cancer, comparing RT with prostatectomy in a recent study in 80,000 individuals. Several studies have demonstrated that the risks for radiation-induced rectal cancer are in particular present after 10 years of follow-up, whereas our median follow-up is currently limited to 8 years with a maximum of 11 years. Furthermore, national screening programs for colorectal cancer might have had an impact on incidences especially in the general population reference group.

With respect to the significantly increased non-pelvic SPC risks for IMRT, we observed several significant results for sub-sites: rectum, urinary tract, and central nervous system (**Table 3**). However, to draw firm conclusions on elevated risks for specific sub-sites using IMRT, we need a larger study population from multiple RT centers, which we are aiming for in a next step of our project.

A major limitation of previous cohort studies investigating the risk of developing SPC in PCa patients is that smoking data are not available from national registry databases and therefore the potential bias of smoking cannot be estimated in such studies. In our study, we were able to assess the impact smoking has on the risk of developing SPCs by evaluating SPC risks in an IMRT and 3DCRT cohort with known and similar distributions of smoking status. We observed a significantly increased incidence of SPCs within the IMRT cohort, compared to the 3DCRT cohort for active smokers whereas for other smoking categories estimated risks were modest. Previous studies have indicated that smoking and RT are interactive factors impacting the risk of developing SPCs, especially for smoking-related cancer sites and in active smokers (18, 29). However, this mechanism is expected in both the 3DCRT and IMRT setting. Based on our results, more scatter dose associated with IMRT combined with active smoking and its associated presence of mutations in organs (30) might have caused additional risks. To our knowledge, this increased risk of SPC in active smokers receiving IMRT has not been reported before, and joined effects of active smoking and peripheral low dose exposure with IMRT have not been modeled before. Further study of this observation is required to draw firm conclusions.

Based on dose measurements and published risk models, several investigators previously hypothesized that modern radiation techniques might be associated with increased SPC risks in PCa survivors (1, 2, 11, 31). A shared view in these studies is that age and beam-on time are key factors. Hall et al. (1), hypothesized that with IMRT a total of about 0.75% additional PCa survivors will develop a SPC compared to conventional RT. Kry et al. (2), estimated this risk as 2–3 times higher in a worst case situation of young patients with long survival. Stathakis et al. (11), calculated that the risk doubles. Sánchez-Nieto (31) ranked treatment techniques based on NTCP and SPC risks and concluded that IMRT was a superior choice and SPC risks had little impact on the ranking, especially for the elderly. Furthermore, several authors recommend a photon energy of 10 MV for IMRT in PCa as the best trade-off between peripheral neutron exposure (which increases with higher energies) and photon exposure (which increases with lower energies because of an increased beam-on time) (2, 32, 33). In our study, IMRT was delivered with both 10 MV and 18 MV. Our (limited) data did not show increased SPC risks for the 18 MV subgroup.

The major strengths of the current study is that we collected detailed RT data as well as smoking data, and compared IMRT to the general population and an internal 3DCRT reference population. The study lacked however statistical power concerning analyses by SPC site specifically and its follow-up is limited with a maximum of 11 years and median of 8 years. Furthermore, we have currently not investigated possible links between estimated dose levels to anatomical sub-sites at risk for SPC (with IMRT and 3DCRT) and observed SPCs incidences. We have planned to perform such analyses within larger multi-center datasets in the future, e.g. within a nested case-control design. Further research and validation of the current results on modern radiotherapy and SPC risks is currently ongoing in a project with a multi-center setting with prolonged follow-up and more detailed RT characteristics.

In conclusion, IMRT is associated with unfavorable excess SPC risks in patients treated at a relative young age, and active smoking might have an additional negative impact on these risks. Further study of causal relationships between IMRT aspects and excess SPC risks in larger multi-center populations is needed with the goal to keep risks for future patients at a minimum.

## Data Availability Statement

The raw data supporting the conclusions of this article will be made available by the authors, without undue reservation.

## Ethics Statement

The studies involving human participants were reviewed and approved by Medische Ethische Toetsings Commissie (METC), Erasmus MC, Rotterdam, The Netherlands. Written informed consent for participation was not required for this study in accordance with the national legislation and the institutional requirements.

## Author Contributions

WH, MH, KA, and LI contributed to the study design. M-CJ, WH, KA, MD, and KV contributed to data collection and analysis. All authors participated in data interpretation. M-CJ, WH, KA, MD, and MH contributed to writing the manuscript. WH, KA, MD, and KV contributed to supervision and study management. All authors contributed to the article and approved the submitted version.

## Funding

For this project a grant was received from the Dutch Cancer Society (KWF), nr 12009. The funder of the study is a non-profit organization and it had no role in study design, data collection, data analysis, data interpretation, or writing of the report. The corresponding author had full access to all the data and had final responsibility for the decision to submit for publication.

## Conflict of Interest

The authors declare that the research was conducted in the absence of any commercial or financial relationships that could be construed as a potential conflict of interest.
