# Supplementary material for: The Risk of Second Primary Cancers in Prostate Cancer Survivors Treated in the Modern Radiotherapy Era
Source: Front Oncol. 2020 Nov 13;10:605119. doi: 10.3389/fonc.2020.605119 (PMC7691574; doi:10.3389/fonc.2020.605119)
Supplement: Supplementary file 1 [file DataSheet_1.pdf]

Online Supplementary File 1. Cancer site, ICD10 code, and applied tumour grouping for analysis.

Subsite malignant cancer	ICD10 code	Tumor type	Pelvis/nonpelvis	Region
**Lip, oral cavity and pharynx**	C0-C14		Nonpelvis	
**Nasal cavity, middle ear, sinuses**	C30,C31		nonpelvis	
**Larynx**	C32		nonpelvis	
**Eye and adnexa**	C69		nonpelvis	
**Meninges**	C70	Central Nervous System	nonpelvis	
**Brain**	C71	Central Nervous System	nonpelvis	
**Pineal Gland**	C75	Central Nervous System	nonpelvis	
**Spinal cord/other parts CNS**	C72	Central Nervous System	nonpelvis	
**Thyroid gland**	C73		nonpelvis	
**Esophagus**	C15	gastrointestinal	nonpelvis	chest
**Stomach**	C16	gastrointestinal	nonpelvis	abdomen
**Small Intestine**	C17	gastrointestinal	nonpelvis	abdomen
**Colon**	C18	gastrointestinal	nonpelvis	abdomen
**Appendix**	C18.1	gastrointestinal	nonpelvis	abdomen
**Rectum + rectosigmoid**	C19,C20	gastrointestinal	pelvis	pelvis
**Anus, anal canal**	C21	gastrointestinal	pelvis	pelvis
**Liver, gallbladder & billiary tract**	C22-C24	gastrointestinal	nonpelvis	abdomen
**Pancreas**	C25	gastrointestinal	nonpelvis	abdomen
**Penis**	C60	genitourinary tract	pelvis	pelvis
**Testis & other male genitalia**	C62,C63	genitourinary tract	pelvis	pelvis
**Kidney**	C64	genitourinary tract	nonpelvis	abdomen
**Renal pelvis**	C65	genitourinary tract	nonpelvis	abdomen
**Bladder**	C67	genitourinary tract	pelvis	pelvis
**Ureter**	C66	genitourinary tract	nonpelvis	abdomen
**Urethra**	C68	genitourinary tract	pelvis	pelvis

**Trachea**	C33		nonpelvis	chest
**Lung & bronchus**	C34		nonpelvis	chest
**Heart, mediastinum, pleura**	C38		nonpelvis	chest
**Pleuramesothelioma**	C38.4		nonpelvis	chest
**Thymus**	C37		nonpelvis	chest
**Breast**	C50		nonpelvis	chest
**Bone limbs**	C40		nonpelvis	
**Pelvic bones**	C41.4		pelvis	pelvis
**Other bones**	C41.0/1/2/3/8/9		nonpelvis	
**Kaposi sarcoma**	C46	soft tissue	nonpelvis	
**Peripheral nervous system**	C47	soft tissue	nonpelvis	
**(Retro)peritoneum**	C48	soft tissue	nonpelvis	abdomen
**Oher soft tissue - headneck**	C49.0	soft tissue	nonpelvis	
**Oher soft tissue - limbs**	C49.1, C49.2	soft tissue	nonpelvis	
**Oher soft tissue - thorax**	C49.3	soft tissue	nonpelvis	chest
**Oher soft tissue - abdomen**	C49.4	soft tissue	nonpelvis	abdomen
**Oher soft tissue - pelvis**	C49.5	soft tissue	pelvis	pelvis
**Other soft tissue - unspecified/overlap**	C49.6-C49.9	soft tissue	nonpelvis	
**Unknown primary -description pelvis**	C80		pelvis	pelvis
**Unknown primary - unclear/outside pelvis**	C80		nonpelvis	

Online Supplementary File 2. Estimated sHRs (sensitivity analysis) when other treatment-related factors are added to the baseline model (age, calendar period, technique) for the primary endpoint of non-pelvic second primary cancer. Abbreviations: sHR = subhazard ratio, CI=confidence interval.

	Fine & Gray Model		
**Treatment characteristic**	**sHR additional factor (95% CI)**	**p-value**	**Estimated sHR for IMRT vs 3DCRT**
Baseline model	-		1.56
+Seminal vesicle dose ≥50 Gy vs 0 Gy	0.97 (0.66-1.42)	0.9	1.56
+Prostate dose 78 Gy vs 72 Gy	0.94 (0.58-1.54)	0.8	1.57
+Hormonal therapy yes vs no	0.90 (0.66-1.22)	0.5	1.57
+Setup verification online vs offline	1.04 (0.59-1.86)	0.9	1.52
Baseline model for 78 Gy subgroup only			1.53
